# Proteomic profile of extracellular vesicles released by *Lactiplantibacillus plantarum* BGAN8 and their internalization by non-polarized HT29 cell line

**DOI:** 10.1038/s41598-020-78920-z

**Published:** 2020-12-11

**Authors:** Svetlana Sokovic Bajic, Maria-Alexandra Cañas, Maja Tolinacki, Josefa Badia, Borja Sánchez, Natasa Golic, Abelardo Margolles, Laura Baldomá, Patricia Ruas-Madiedo

**Affiliations:** 1 Department of Microbiology and Biochemistry of Dairy Products, Instituto de Productos Lácteos de Asturias – Consejo Superior de Investigaciones Científicas (IPLA-CSIC), Villaviciosa, Asturias Spain; 2 Group Funcionality and Ecology of Beneficial Microbes, Instituto de Investigación Sanitaria del Principado de Asturias, (ISPA), Oviedo, Asturias Spain; 3 Laboratory for Molecular Microbiology, Institute of Molecular Genetics and Genetic Engineering (LMM-IMGGE), University of Belgrade, Belgrade, Serbia; 4 Secció de Bioquímica I Biología Molecular, Departament de Bioquímica I Fisiologia, Facultat de Farmàcia I Ciències de L’Alimentació, Universitat de Barcelona, Barcelona, Spain; 5 Institut de Biomedicina de la Universitat de Barcelona (IBUB), Institut de Recerca Sant Joan De Déu (IR-SJD), Barcelona, Spain; 6 Present Address: Laboratorio de Endocarditis Experimental, Hospital Clinic – Institut d’Investigacions Biomèdiques August Pi i Sunyer (IDIBAPS), Barcelona, Spain

**Keywords:** Bacterial host response, Bacterial physiology, Bacterial structural biology

## Abstract

In recent years the role of extracellular vesicles (EVs) of Gram-positive bacteria in host-microbe cross-talk has become increasingly appreciated, although the knowledge of their biogenesis, release and host-uptake is still limited. The aim of this study was to characterize the EVs released by the dairy isolate *Lactiplantibacillus plantarum* BGAN8 and to gain an insight into the putative mechanism of EVs uptake by intestinal epithelial cells. The cryo-TEM observation undoubtedly demonstrated the release of EVs (20 to 140 nm) from the surface of BGAN8, with exopolysaccharides seems to be part of EVs surface. The proteomic analysis revealed that the EVs are enriched in enzymes involved in central metabolic pathways, such as glycolysis, and in membrane components with the most abundant proteins belonging to amino acid/peptide ABC transporters. Putative internalization pathways were evaluated in time-course internalization experiments with non-polarized HT29 cells in the presence of inhibitors of endocytic pathways: chlorpromazine and dynasore (inhibitors of clathrin-mediated endocytosis—CME) and filipin III and nystatin (disrupting lipid rafts). For the first time, our results revealed that the internalization was specifically inhibited by dynasore and chlorpromazine but not by filipin III and nystatin implying that one of the entries of *L. plantarum* vesicles was through CME pathway.

## Introduction

Archaea, Bacteria and Eukarya produce and release extracellular vesicles (EVs) that are membrane-contained structures playing a relevant role in the inter-communications established among these tree domains of life; however, there are still many unsolved questions on this field^1^. Firstly, nomenclature is sometimes confusing. In eukaryotic cells it can be broadly classified as: microvesicles (microparticles or ectosomes) that are produced from the cytoplasmatic membrane; exosomes, intracellularly formed through endosomes and released upon fusion with the cytoplasmatic membrane; and apoptotic bodies which are released from apoptotic cells^2^. In the case of bacteria, most studies have been performed with Gram-negatives and two types of bacterial EVs have been described: outer membrane vesicles (OMVs), derived from outer membrane that enclose periplasmic components, and outer-inner membrane vesicles (O-IMVs) formed by the protrusion of both, outer and cytoplasmic, membranes that could be enriched in cytoplasmic components^3,4^. Extracellular vesicles in Gram-positive bacteria have been purified more recently, and mostly in pathogenic species^5,6^. They are collectively referred as bacterial EVs or membrane vesicles (MV); however some authors propose the name of cytoplasmic (CMVs)^7^, whereas others use the term MV to refer to empty vesicles, for example right side-out and inside-out MV from *Escherichia coli* applied for studying mechanism of transport in different bacteria^8^. In this work, dealing with the characterization of vesicles produced by *Lactiplantibacillus plantarum* (formerly *Lactobacillus plantarum*), we will use the term EVs.

The biogenesis and release of bacterial EVs is not well understood. In Gram negatives two mechanisms have been proposed: “blebbing of” membrane material of living cells and endolysin-triggered cell lysis^7^. For Gram-positives the picture is even more unclear, and most studies have been carried out in *Bacillus subtillis* and *Staphylococcus aureus*, since the release of EVs seems to be involved in their pathogenicity^7,9^. It has been proposed that an endolysin (prophage encoded) triggers the protrusion and release of EVs through a hole in the peptidoglycan of *B. subtilis* which, although does not provoke its lysis, weaken the cells due to the loss of membrane integrity^10^; this process is known as “bubbling cell death” and is not exclusive of this bacterium^7^. In the case of *S. aureus*, phenol-soluble modulins are required for the release of EVs because these peptides facilitate an increase in the fluidity of the cytoplasmic membrane^11^. Indeed, the modulins are also involved in the recognition of EVs by TLR (toll-like-receptor)-2 located in the surface of the host cells, which is one of the mechanisms described for bacterial EVs uptake and internalization^12,13^.

One of the intriguing questions that had promoted a lot of research is what the bacterial EVs carry out inside. In fact, thinking from a human perspective, they have been proposed as “cargo” structures that harbour different molecules of pivotal relevance for the interaction with the host, both in negative (pathogenesis) or positive ways^14^. Just to summarize some of the most recent studies, it has been showed that EVs released by probiotics are able to interact with pattern recognition receptors (PRR) of host cells initiating the downstream immune response modulation which, contrary to that induced by pathogens (pro-inflammatory) is more tolerogenic^15–18^. Additionally, EVs released by *L. plantarum* were able to in vivo regulate the brain function being able to reduce the stress-induced depression in mice^19^. Therefore, the EVs released by probiotics, or by the intestinal microbiota, could have multiple applications on human health^6,20,21^. Indeed, bioengineering bacterial EVs produced by commensal bacteria has been proposed as a putative method for the delivery of biotherapeutics to the intestinal tract^22^. Finally, another open question in this field, related to the purification of bacterial EVs within a pool of eukaryotic EVs from human body fluids has been recently addressed^23^, which will open an avenue for research on the application of these cargo vehicles.

Recently, it has been reported the isolation and proteomic characterization of *Lactobacillus*-derived EVs vesicles from three strains of the probiotic species *L. casei*, *L. acidophilus* and *L. reuteri*^24^. The aim in the current work is to achieve the characterization of the EVs released by the potential probiotic *L. plantarum* BGAN8, an exopolysaccharide (EPS) producing strain, as well as, given the scarce information about the internalization pathways in Gram-positives, to gain insight about the putative mechanism of probiotic EVs uptake by intestinal epithelial cells.

## Results

### Microscopic analyses

**Figure 1:**
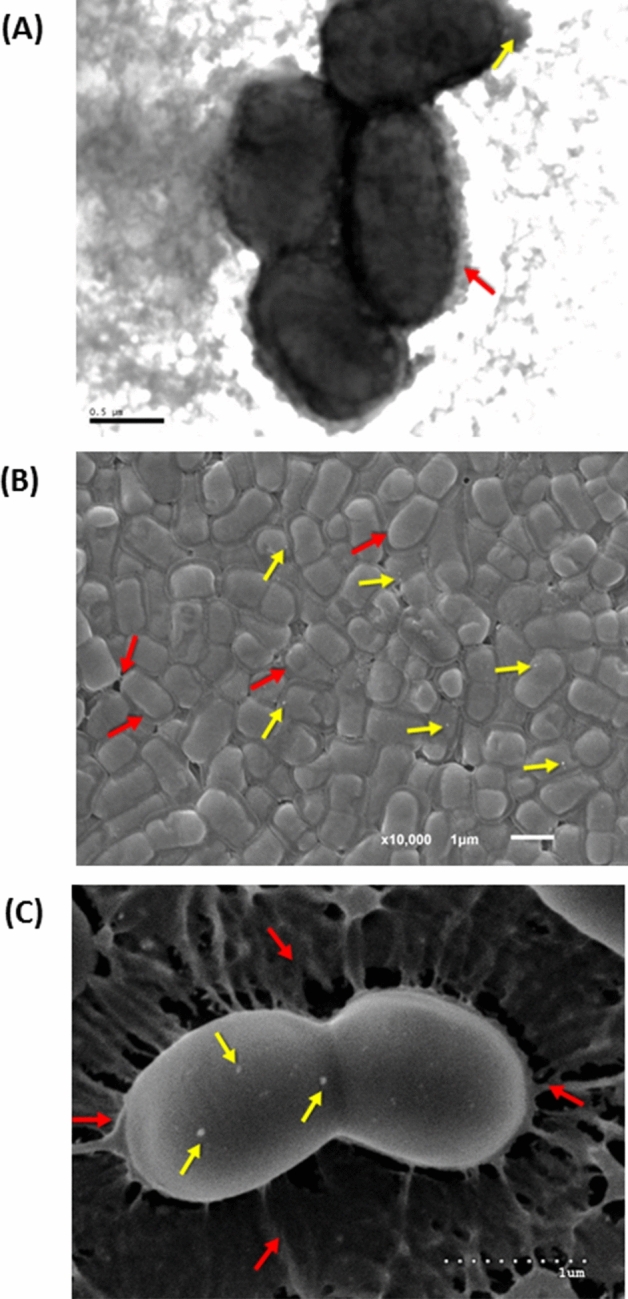
Visualization of *L. plantarum* BGAN8 by different electron microscopy techniques. **(A)** Transmission electron microscopy (bar 0.5 μm). (**B**) Scanning electron microscopy (bar 1 μm). (**C**) Cryo-scanning electron microscopy (bar 1 μm). The red arrows show the EPS covering the surface of the strain and the yellow arrows underline the presence of EVs. For additional microphotographs see supplementary material.

The observation of *L. plantarum* BGAN8 using different microscopy techniques revealed the presence of EVs in its surface as showed in Fig. 1 (see supplementary Figs. S1, S2 and S3). The presence of an EPS layer surrounding the cell somehow difficult the detection of a defined contour of the exocellular formation by means of TEM (Fig. 1A), contrary to that observed for other strains^25^. However, in a closer view of the bacterial surface (Fig. S1) it was possible to detect a rounded protrusion, darker than the EPS layer and showing a rough aspect, probably, due to the presence of the polymer; the size seems to be smaller than 100 nm. The SEM images obtained (Fig. 1B, Fig. S2) also denoted the presence of small “dots”, being some of them shinier than the rest of the surface^25^. Nevertheless, the cryo-SEM observation, the less invasive technique that only involves the liquid N_2_ frozen of the bacterial suspension, undoubtedly revealed the presence of small EVs in the surface of this EPS-producing strain, as well as clearly showed the presence of a polymer loosely attached to the cell (Fig. 1C, S3).

**Figure 2:**
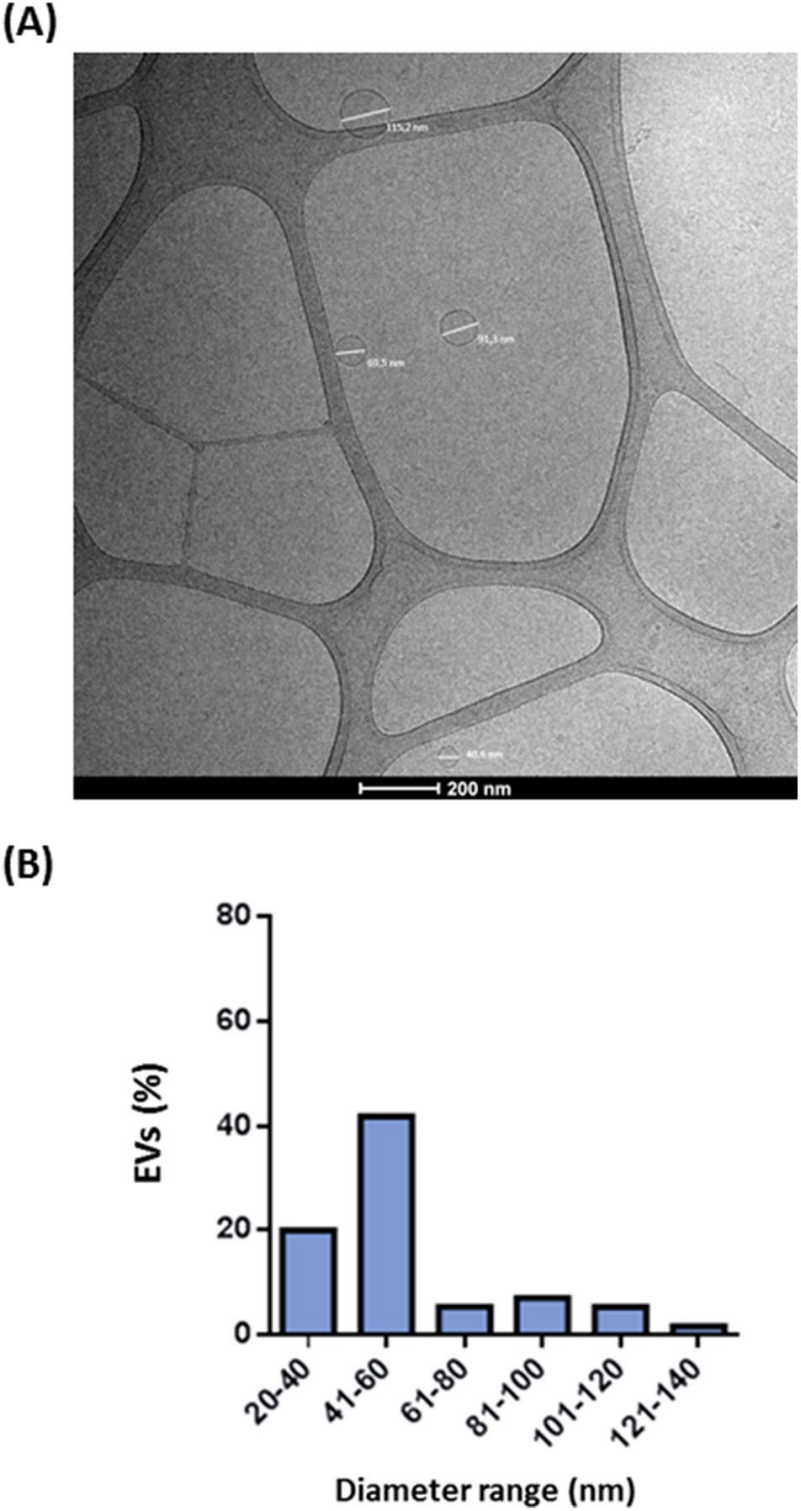
Extracellular vesicles purified from *L. plantarum* BGAN8 cell-free supernatants. (**A**) EVs visualized by cryo-transmission electron microscopy (bar 200 nm); size of the particles (from up to bottom and left to right): 115.2 nm, 69.5 nm, 91.3 nm and 40.6 nm). (**B**) Size distribution of the EVs according to the diameter range (nm) measured under the cryo-TEM microscope.

The next approach was to purify this material from viable BGAN8 cultures (supplementary Fig. S8) using standard procedures described in literature. The pellet obtained (Fig. S4) resembles that previously reported^26^ but, in our case, the EVs sediment obtained after ultracentrifugation was very viscous, thus suggesting the co-precipitation of part of the tightly EPS-attached layer, as it was pointed in the TEM analysis (Fig. S1). To check the presence of EVs, this pellet was submitted to cryo-SEM analysis; however, the small size of the particles, very near to the resolution level of the equipment used, and the presence of the EPS in the sample difficult the obtaining of good resolution images. Nevertheless, it was possible to detect an EPS network with small spherical particles imbed inside; this suggests that the polymer also form part of the surface of the EVs (Fig. S5). Finally, the cryo-TEM technique was applied as a close-to-native state visualization due to minimal sample manipulation. EVs samples were vitrified by rapid immersion in ethane and directly visualized by cryo-microscopy at liquid nitrogen temperatures in the absence of any added contrast agent^3^. This analysis revealed the presence of a single layer of EVs that can be found as separate structures (Fig. 2A) or as small groups of vesicles trapped in an undefined network of, probably, EPS (Fig. S6). The EVs of *L. plantarum* BGAN8 displayed a homogeneous, spherical morphology with size that ranged from 20 to 140 nm. Indeed, the size distribution of EVs (Fig. 2B) showed the highest abundance (43.8%) in the range between 41 and 60 nm, followed by 20–40 nm (about 20%), and the remaining (about 36%) with a bigger size (between 61 and 140 nm). In spite of the scarce available data, we consider that this size distribution is similar to that reported in literature for bacterial EVs of lactobacilli strains^25–28^, specifically for *L. plantarum*^19,29^, whereas the reported range of EVs from pathogenic Gram-positive bacteria seems to be slightly higher, between 10 and 400 nm^6^.

### Proteomic analyses

**Figure 3:**
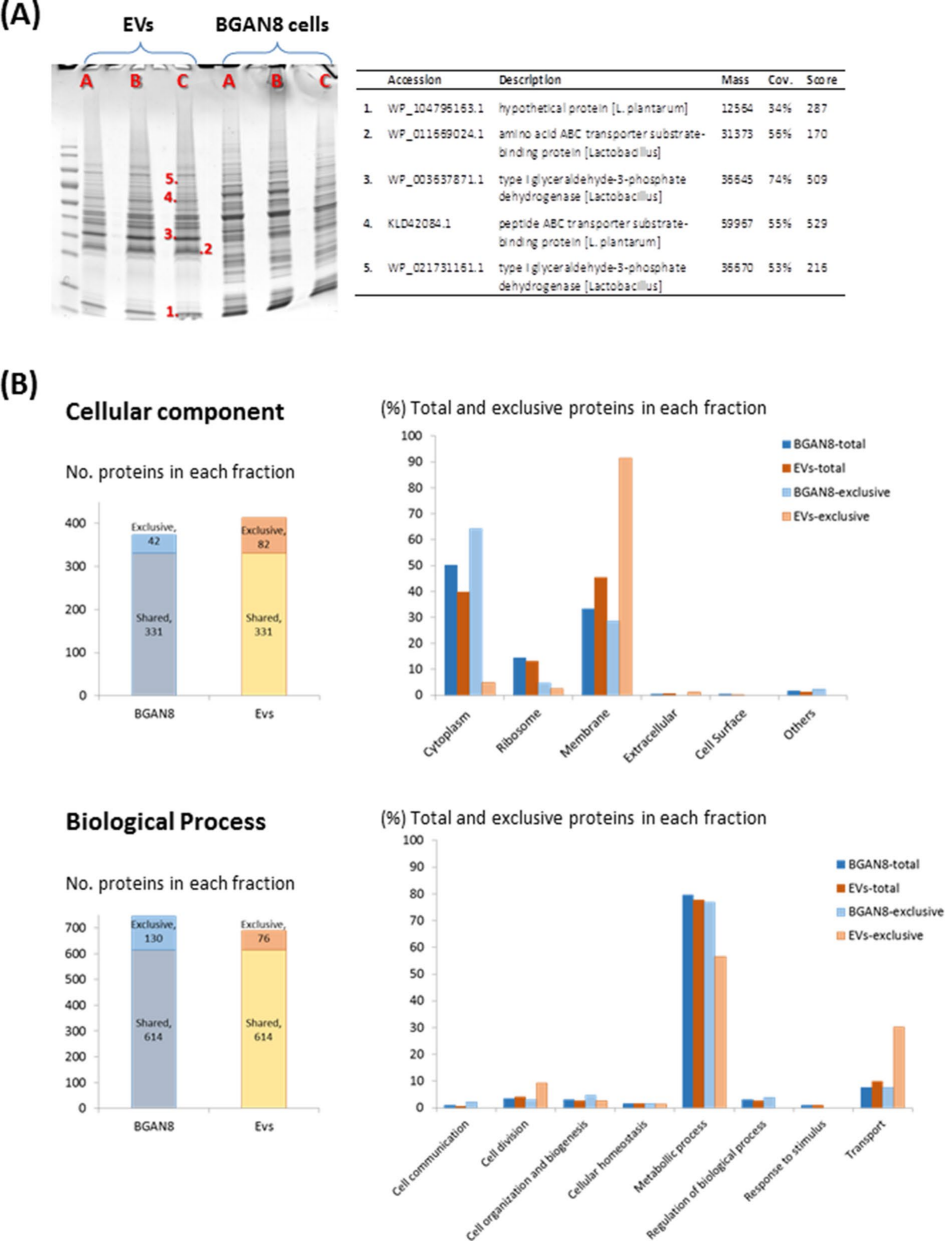
Proteomic fingerprint of the whole cell-extract and the EVs from *L. plantarum* BGAN8. (**A**) SDS-PAGE profile of three culture replicates (A, B, and C) of EVs and BGAN8 whole-extract. Protein identification (table) of some excised bands (1 to 5) was performed by a MALDI-TOF mass spectrometer and comparison against the NCBIprot non-redundant database. (**B**) Peptide fingerprint analysis performed by liquid chromatography and mass spectrometry. After protein identification (see supplementary data file ), the proteins with assigned functions “cellular component” and/or “biological process” were analysed and results presented here.

To undertake a preliminary characterisation of the *L. plantarum* BGAN8 EVs, its proteomic profile was analysed. Firstly, a SDS-PAGE study of EVs extracted from supernatants of three independent cultures, and the corresponding whole protein fractions obtained from the bacterial pellets, was performed. Results obtained revealed a different protein profile of EVs with respect to the whole protein content of the bacterium, being the three replicates very reproducible among them (Fig. 3A). Five major EV protein bands (identified in the SDS-PAGE gel from 1 to 5) were excised, analysed by MALDI-TOF mass spectrometry and identified by comparison against the NCBIprot non-redundant database, resulting in the identified proteins presented in the Table of Fig. 3A. It is noticeable that the most abundant bands corresponded with amino acid / peptide ABC transporters (bands 2 and 4) and with type I glyceraldehyde-3-phosphate dehydrogenase (GAPDH, bands 3 and 5). These suggest that the EVs are enriched in membrane components as well as enzymes involved in central metabolic pathways such as glycolysis. In this regard, it has been previously reported the “moonlighting” function (or multifunctionality) of some of glycolytic enzymes which can be present in the surface of lactobacilli thus being involved in other physiological events; in the case of GAPDH, it could be involved in the adhesion of *L. plantarum* to the intestinal epithelium^30^.

Next, the triplicates of each fraction (bacterial pellet or EVs) obtained from three independent cultures of *L. plantarum* BGAN8 were mixed and analysed by means of LC–MS/MS for peptide profiling and further protein identification. Results obtained are available as supplementary material (see Database of protein fingerprint.xlsx). The number of total proteins identified was 1387 of which, 979 where common in both fractions whereas 238 where exclusively present in the BGAN8 whole-fraction and 170 in the EVs fraction. Of the total proteins, biological functions were assigned for about 59% (820 proteins), whilst the subcellular location (cellular component) was assigned for approximately 33% (455 proteins). Figure 3B shows a summary of the cellular component and the biological process of proteins with these two characteristics assigned after the analysis of the raw data (see supplementary methods for details). Regarding the cellular location, 331 proteins were shared between both fractions and, remarkably, 82 were exclusively present in the EVs fraction while 42 were only found in the whole protein bacterial one (Fig. 3B, left panel up). It is noticeable that, among the proteins with cellular location assigned and exclusively present in the EVs (Fig. 3B, right panel up), most of them by far (91.5%) corresponded with a membrane location, whereas 28.6% had a membrane location in the proteins exclusively found in the whole protein fraction. This could be expected taking into account the putative way of bacterial EV formation and release through the cell wall^5^. In the case of the whole bacterial-fraction, most of the exclusive proteins corresponded with those located in the cytoplasm (64.3%). The analysis of the proteins with biological process assigned showed that 614 were common between both fractions and, in this case, the whole bacterial-fraction presented higher number (130) of exclusive proteins with known function than those (76) found only in the EVs fraction (Fig. 3B, left panel down). Of the exclusive assignation, most of the proteins were involved in metabolic processes (76.9% and 56.6% for whole bacterial-fraction and EVs, respectively) (Fig. 3B, right panel down); but, the EV fraction was near 4-times richer in proteins related to transport functions than the whole-extract (30.3% and 7.7%, respectively). The coherent results obtained, either regarding the cellular location or the biological function, support the fact that EVs released by *L. plantarum* BGAN8 were mainly enriched in membrane components with a high fraction of proteins involved in transport.

### Internalization by HT29 cell line

Bacterial EVs have been proposed as delivery cargo of molecules involved in the bacteria-gut interaction, which could play a role on intestinal health and homeostasis^6^. Given that the intestinal epithelium is the first physical barrier that gut bacteria encounter, we have tested the response of HT29-cell monolayers in the presence of UV-inactivated *L. plantarum* BGAN8 pellets or different concentrations (determined by protein content) of the purified EVs. The real time monitoring of HT29 evolution after these treatments did not show any noticeable change in the impedance signal, thus indicating that there are no toxic effects nor detectable morphological and/or membrane-activity changes (Fig. S7).

**Figure 4:**
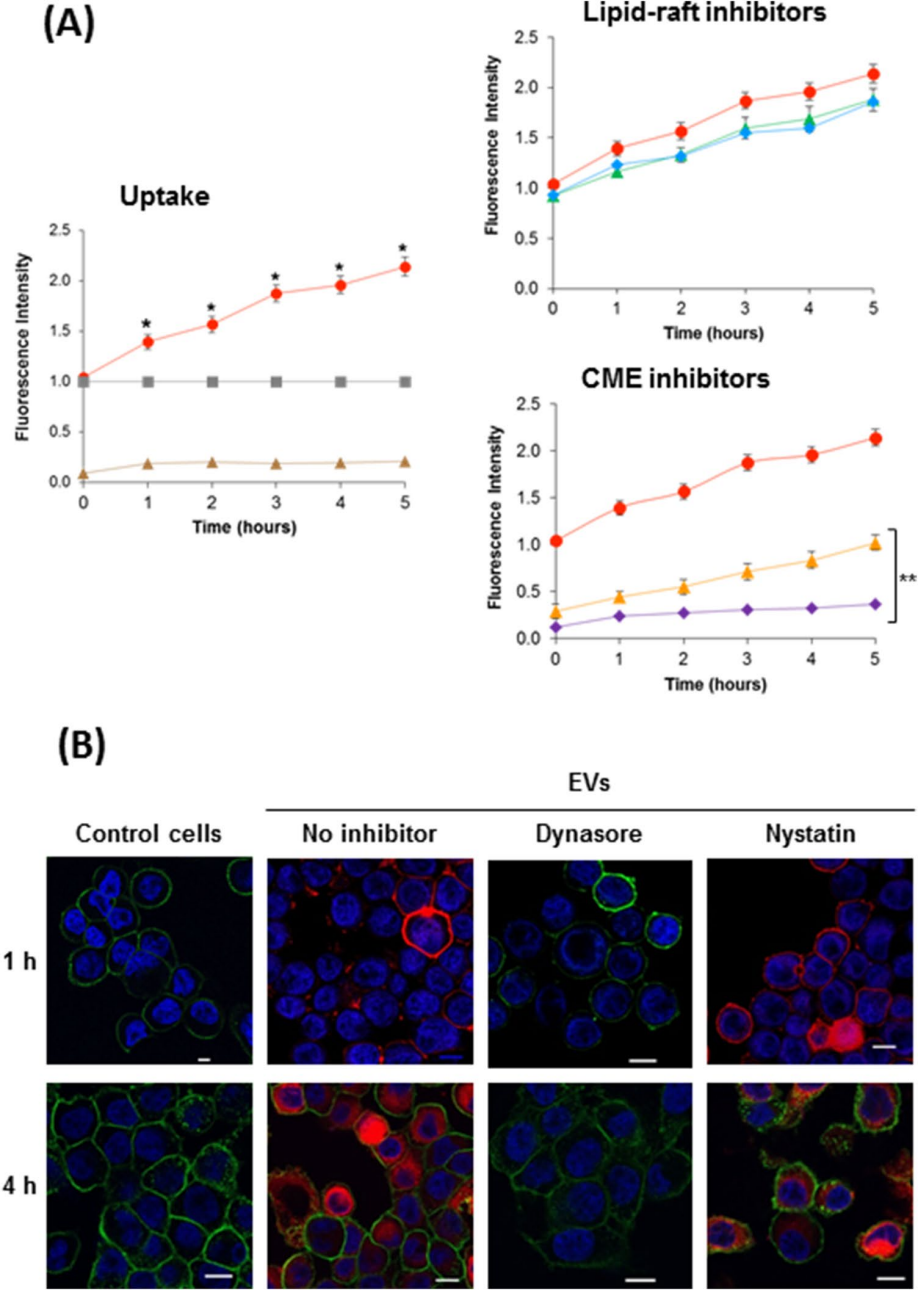
Inhibitors of clathrin-mediated endocytosis block internalization of *L. plantarum* EVs in HT29 cells. (**A**) The left panel shows the vesicle uptake in the absence of endocytosis inhibitors: HT29 cells incubated with rhodamine B-R18-labeled EVs (1 μg/well) from *L. plantarum* (red circles); controls: EVs (grey squares) and HT29 cells (brown triangles). The right panels show the uptake of *L. plantarum* EVs in the presence of inhibitors of endocytosis pathways: HT29 cells pre-incubated (prior to the addition of rhodamine B-R18-labeled EVs) with the lipid raft disrupting agents filipin III (blue diamonds) or nystatin (green triangles), or with the clathrin-mediated endocytosis (CME) inhibitors chlorpromazine (yellow triangles) or dynasore (purple diamonds). The uptake in the absence of inhibitors is shown as a control (red circles). Fluorescence intensity was normalized by fluorescence detected at the indicated time points by labelled EVs in the absence of HT29 cells. Data are presented as means ± standard error from three independent experiments. Statistical differences were assessed using one-way ANOVA followed by Tukey’s test: *, significance against untreated control cells (*p* ≤ 0.001); **, significance against uptake values in the absence of inhibitors (*p* ≤ 0.012). (**B**) Visualization of internalized EVs by confocal fluorescence microscopy in the absence (no inhibitor) and the presence of the inhibitors dynasore and nystatin after 1 h and 4 h of incubation. Control cells: HT29 in the absence of EVs. Nuclei were stained with DAPI (blue), the cell membrane with WGA-Alexa Fluor-488 (green) and internalized EVs with rhodamine B-R18-label (red). Scale bar: 20 μm.

In order to check whether the EVs from *L. plantarum* BGAN8 were able to interact with these enterocyte-like cells, putative internalization pathways were evaluated using a spectrofluorometric technique. To do that, HT29 monolayers (at least 80% confluence) were incubated with rhodamine B-R18-labeled EVs (1 μg/well), and the kinetics of EVs uptake were monitored over 5 h (Fig. 4A). Results indicated that *L. plantarum* EVs were internalized in HT29 cells since a time-dependent increase in fluorescence was observed when the enterocytes were incubated with EVs, but not in the control cells (no EVs added) or rhodamine-labelled EVs alone (no HT29 presence) (Fig. 4A, left panel). To identify the entry pathway of *L. plantarum* EVs in HT29 cells, time-course internalization experiments were performed in the presence of inhibitors of several endocytic pathways. Specifically, the cholesterol-sequestering agent’s filipin III and nystatin were used to disrupt lipid rafts microdomains and caveolae, whereas chlorpromazine and dynasore were used to inhibit clathrin-mediated endocytosis (CME). HT29 cells were pre-incubated for 1 h with these inhibitors and, afterwards, lactobacilli EVs uptake was measured by the increase in the fluorescence emitted by HT29 cells upon rhodamine-labelled EVs addition. Internalization was inhibited by chlorpromazine and dynasore but not by filipin III or nystatin (Fig. 4A, right panels). These results support that entry of *L. plantarum* vesicles was through CME and that cholesterol-enriched lipid rafts seems not to be required for this process.

Additionally, internalization of *L. plantarum* EVs by HT29 cells was also analysed by confocal fluorescence microscopy after 1 and 4 h of co-incubation. To confirm the internalization route, parallel assays were performed in the presence of dynasore and nystatin. For these studies, rhodamine-labelled EVs were used at 1 μg/well (red), HT29 nuclei were labelled with DAPI (blue) and plasma membranes with WGA (green) (Fig. 4B). In the absence of endocytosis inhibitors, EVs were visualized mainly adhered to the surface of HT29 cells after 1 h incubation; at this time the green signal of the HT29 membranes was not observed, probably because EPS-trapped vesicles adhered to the cell membrane impaired proper visualization or labelling of the HT29 membrane. After 4 h-incubation, the red fluorescence was observed inside the cytoplasm suggesting that *L. plantarum* EVs were taken up by HT29 cells; at this time, the HT29 membrane was visualized given that EPS would not interfere with the signal (Fig. 4B, no inhibitor). Microscopy analysis also confirmed the effect of endocytosis inhibitors on EVs internalization. As shown in Fig. 4B, internalization of rhodamine-labelled EVs in HT29 cells was specifically inhibited by dynasore (no red signal inside the cytoplasm), whereas nystatin had no effect. These results are consistent with the CME pathway demonstrated by the spectrofluorometric technique.

## Discussion

*Lactiplantibacillus plantarum* BGAN8 is a natural isolate from an artisanal cheese originated from Andrijevica municipality, eastern Montenegro. It was selected for this study due to its probiotic potential and to the safety QPS status (Qualified Presumption of Safety) of this species listed by the EFSA (European Food Safety Authority; EFSA)^31^. Among the characteristics that could be involved in its health benefits, the synthesis of an EPS as well as the production of γ-aminobutyric acid (GABA) (unpublished data) could be underlined. In this regard, it has been previously reported that EPSs produced by LAB, such as some lactobacilli species, and bifidobacteria have been associated with probiotic attributes^32–34^, while our recent data indicated that GABA producing lactobacilli play a significant role in reduction of inflammation^35^. Here we aimed to further characterize this strain studying its capability to in vitro release EVs and to interact with intestinal epithelial cells as putative mechanisms involved in the bacterial-host cross-talk.

### *L. plantarum* BGAN8 release EVs enriched in membrane transporters

In this work we have demonstrated that the EPS-producing *L. plantarum* BGAN8 strain is able to release extracellular vesicles after 20 h of cultivation in MRS, which corresponded with early stationary phase, being the polymer one of the components of the vesicles. The most valuable technique to visualize the presence of these EVs, both purified or located in the surface of the bacteria, involved the use of cryo-electron microscopy (cryo-TEM and cryo-SEM) in which the samples were visualized almost intact after liquid N_2_ congelation. In addition, the cryo-SEM allowed demonstrating the production of EPS which can be easily lost during the fixation, and centrifugations steps required for other electron microscopy techniques^36^. On the other hand, the cryo-TEM is a powerful tool to distinguish a large variety of EVs with different morphology and sizes^37^, including OMVs from Gram negative bacteria^3,38^ or MVs from Gram-positives, such as *Streptococcus pneumoniae*^39^; as far as we could find, this is the first time that lactobacilli EVs are visualized using this technique. Additionally, the good resolution of cryo-TEM images allowed the size quantification of the EVs. For *L. plantarum* BGAN8 the vesicle size was variable, predominantly in the 20–60 nm range. When comparing with other bacterial EVs, it is worth noting that the size calculation is highly dependent on the measurement method. In any case, variable size distribution of extracellular vesicles have been reported for Gram-positive bacterial EVs other than lactobacilli^40^, but also for a number of *Lactobacillus* species^16,24–27,29,41,42^. Given the scarce literature, it cannot be postulated if the size distribution of lactobacilli EVs could be a strain-specific or a species-associated characteristic and whether culturing or environmental conditions could influence the release of these structures.

The proteomic analysis carried out showed that most proteins found in the EVs released by *L. plantarum* BGAN8 were predicted to be located in the membrane (Fig. 3B: 45.3% for EVs-total) which is the main location found in the exclusive fraction of the vesicles (91.5%). In the case of the whole bacterial extract, both total and exclusive fractions, proteins with cytoplasmic location were the most abundant (50.1% and 64.3%, respectively). Membrane-enriched and cytoplasmic-depleted subcellular locations were also found in the proteomic characterization of OMVs from *Pseudomonas aeruginosa*^43^ and *Escherichia coli*^44^. In the case of Gram-positives, extracellular (cell wall, membrane or secreted) and periplasmic location constituted the 63% of the identified proteins (vs. 47% cytoplasmatic) in EVs from *Streptococcus suis*^45^. Li and co-workers^29^ found that over half of the proteins present in EVs released by *L. plantarum* WCFS1 were found to be associated with membrane. Elevated levels of secreted, cell-wall associated and membrane categorised proteins in EVs released from three strains of *Lactobacillus acidophilus, L. casei* (*Lacticaseibacillus casei*) and *L. reuteri* (*Limosilactobacillus reuteri*) were also reported^24^. On the contrary, in the *L. casei* BL23 strain most of the proteins of the EVs were reported to have cytoplasmatic location^26^. This feature could be related to the distinct cargo molecules that EVs released from different species could harbour^4^, or to the specificity and accuracy of the different EVs isolation methods. In fact, the capability of EVs from *Lactobacillus crispatus* BC3 and *Lactobacillus gasseri* BC12 to ex vivo protect vaginal tissues from HIV-1 infection was due to the presence of specific proteins and/or metabolites able to inhibit the virus, since this effect was not observed for EVs released from other vaginal lactobacilli^42^. Apart from the cellular location, proteins participating in a broad array of biological processes, such as cell division, communication or biogenesis, cellular homeostasis or regulation, but, mainly, metabolic pathways and transport, were identified in the EVs from *L. plantarum* BGAN8 (Fig. 3B). As expected, similar functional classification was found in the proteome of other bacterial extracellular vesicles, in spite of the different approaches for data analysis^24–26^. The EVs from *L. plantarum* BGAN8 are enriched in proteins associated with transport events. An in-deep analysis of the 43 proteins exclusively detected in EVs located in the membrane with assigned function, showed that 17 were involved in transport; eight of them (Numbers: 449, 745, 841, 886, 1203, 1250, 1274 and 1300; see supplementary data file) are predicted to be amino acid/oligo-peptide transporters. This supports the results obtained from SDS-PAGE since bands 2 and 4 (Fig. 3B amino acid/peptide ABC transporters) were highly enriched in the EVs extract. The remaining transporters exclusively found in the EVs are predicted to be involved in the transport of glycerol (Number 987), niacin (Number 1387) and nucleosides (Numbers 1018 and 1270) or are permease-like proteins (Numbers 843, 1143, 1152 and 1303). Interestingly also a protein identified as belonging to a polysaccharide biosynthesis family [Number 1125, Pfam IDs: f01943 (integral membrane protein implicated in production of polysaccharide), Pf03023 (lipid II flippase), Pf14667 (C-terminal integral membrane region of polysaccharide biosynthesis proteins)] was identify in the EVs of this EPS-producing strain. This protein could be part of the Wzx (flippase-like protein)—Wzy (polymerase) system that translocates the lipophilic-carrier linked EPS-repeating unit of monosaccharides across the cell membrane to build the polymer^32^. Then, this could support the microscopic observation that EPSs synthesized by *L. plantarum* BGAN8 seem to be a component of their EVs. Nevertheless, the presence of EPSs as structural component of the EVs released by the lactobacilli must be proven with additional experimentation procedures.

### Inhibitors of clathrin-mediated endocytosis block the internalization of *L. plantarum* EVs in HT29 cells

In non-phagocytic cells the entrance of small molecules across the membrane bilayer of a cell is achieved by four mechanisms: macropinocytosis, clathrin-mediated endocytosis (CME), caveolin-mediated endocytosis, and non‐caveolin/non-clathrin-mediated endocytosis (using lipid rafts or direct membrane fusion); all of them have been described as pathways for the uptake of OMVs from Gram-negative bacteria^13^. In fact, OMVs internalization is mainly driven by endocytosis and the specific pathway depends on the surface and cargo of the vesicles^46^. To the best of our knowledge, the pathway(s) involved in the uptake of EVs secreted by *Lactobacillus* spp. has not been reported so far^6^. In this work we have first showed that intestinal epithelial cells are able to internalize EVs released from *L. plantarum* BGAN8 and that the uptake kinetics by non-polarized HT29 was slower than that reported for vesicles of Gram-negative probiotics. In the same cellular model, the OMVs from the probiotic strain *E. coli* Nissle 1917 were intracellularly observed at earlier incubation times, less than 1 h^46^, whereas the EVs from *L. plantarum* BGAN8 required more time. The delayed kinetics of EVs entry may be attributed to a trapping effect of the abundant secreted EPS material (see supplementary Fig. S5) that accompanied isolated EVs. Alternatively, the EPS layer that seems to remain in the cover of EVs (see supplementary Fig. S1) may somehow restrict or defer their binding to specific host cell membrane structures required for CME. An elegant way to unequivocally demonstrate whether EPS are forming part of EVs and if in this form are responsible for the delayed uptake by intestinal cells would be the use of isogenic non-EPS producing mutants. However, to date, this has been an unsuccessful strategy given that it was not possible to genetically manipulate this wild type *L. plantarum* strain.

Secondly, we have studied the EVs internalization pathways by using inhibitors of the three endocytic-mediated processes, specifically: filipin III and nystatin that inhibit lipid rafts and caveolae pathways, and chlorpromazine and dynasore that act upon CME, the last one acting upon the protein dynamin required for membrane remodelling and excision of clathrin-coated vesicles^13,47^. Our results showed that the EVs released by *L. plantarum* BGAN8 were internalized by the CME pathway, since no uptake was observed using chlorpromazine and dynasore, whereas EVs were still internalized in the presence of inhibitors directed to lipid rafts. There is not much data available about the uptake mechanism of EVs from Gram-positive bacteria by host cells, although it has been reported that this event occurs, as in the case of *S. pneumoniae* EVs that are internalized by immune cells^39^. The four non-phagocytic mediated mechanisms described above have been reported for the internalization of OMVs released by Gram-negative bacteria into epithelial cells. The most frequent mechanism involves lipid rafts; in addition, the interaction with specific PRR, such as TLR2, also facilitates the entry of OMVs^12,18^. Lipid rafts are micro-domains within the plasma membrane of eukaryotic cells that have more abundance of protein receptors and sphingolipids, which affect its fluidity (less fluid), and are also enriched in cholesterol that is required for endocytosis trafficking^48^. We could hypothesize that the EPSs layer surrounding the EVs of *L. plantarum* BGAN8, together with the presence of a thick cell wall, could hinder the interaction of the bacterial membrane with lipid rafts microdomains. On the contrary, the interaction of the EVs with an unknown receptor could trigger the progressive and sequential assembly of clathrin molecules, forming pits that deform the membrane which finally collapses forming the intracellular vesicle^13,48^. Therefore, our findings show that the internalization of *L. plantarum* EVs is mediated, at least in part, through CME and suggest that it may be somehow modulated by the associated EPS. However, the precise mechanism still remains to be totally elucidated. In fact, certain bacterial EVs have been shown to use more than one pathway to enter epithelial cells depending on the vesicle size (reviewed by O'Donoghue & Krachler^13^). In our case, the EVs purified from cultures of *L. plantarum* BGAN8 are entrapped in a network of EPSs released to the culture medium, which have co-precipitated with the EVs (supplementary Fig. S5). The polymeric material created a big volume layer around the vesicles. Therefore, macropinocytosis process could be also involved in the uptake of these EV gulps. This hypothesis deserves additional experiments to be demonstrated.

## Conclusion

Extracellular vesicles mediate cell–cell interactions within and between cells from different kingdoms. EVs have been extensively studied in eukaryotic cells, where they have been found to perform important functions in modulating physiological conditions. These functions are triggered by the cargo of macromolecules present inside the vesicles, mainly proteins and nucleic acids. However, much less attention has been paid to the study of bacterial EVs, specifically from non-pathogenic Gram-positives. In the last decade there are several works showing that these bacterial structures can carry DNA, RNA, proteins or toxins and play important roles in the communication between bacteria and with host cells, such as gene transfer events, modulation of immunity and contribution to pathogenesis. In relation to this, the experimental work carried out in our study demonstrated that *L. plantarum* BGAN8 produces EVs containing a pool of exclusive proteins which is highly enriched in membrane proteins and in proteins involved in cell processes linked to the cell surface, in comparison with the whole cell protein content. In addition, *L. plantarum* BGAN8 EVs can be taken up by intestinal epithelial cells by more than one pathway. In this context inhibitors of CME inhibited the uptake of these EVs into enterocytes, suggesting that their internalization is carried out through a clathrin-related pathway. Our results also suggest that this uptake could be somehow regulated by the presence of EPS, since this polymer seems to be linked to the vesicle structure. However, more experiments are needed to prove that EPS are truly components of EVs. On the other hand, macropinocytosis may account for the uptake of large vesicle gulps surrounded by secreted EPS. Further studies will also determine the biological processes in which this *L. plantarum* EVs may be involved.

## Methods

### EVs purification

*L. plantarum* BGAN8, belonging to the LMM-IMGGE culture collection, was grown in MRS broth (Difco, BD Biosciences, San Diego, CA) at 30 °C for 20 h to early stationary phase. Growth was monitored by continuous measurement of pH (ORION Versa Star, Thermo Scientific Inc., USA), and by measuring the optical density (OD_600 nm_) and viable bacteria cell counts on agar-MRS at defined points (Fig. S8). Extracellular vesicles (EVs) were isolated as previously described with minor modifications^46^. In brief, BGAN8 grown cultures (1 L each) were centrifuged (10,000*×g*, for 20 min, at 4 °C) to collect bacterial pellets and supernatants. The last were filtrated, through 0.45 μm-pore size cellulose acetate membranes (Merck, Darmstadt, Germany), and concentrated using the Centricon Plus-70 membrane Ultracel-PL, 100 kDa cutoff (Millipore, Merck) following the manufacturer’s instructions. Concentrated supernatants were centrifuged at 260,000*×g* for 1 h at 4 °C and washed twice with phosphate buffered saline solution (PBS), or with 0.1 M phosphate buffer without salts (PB). The sediments containing EVs were resuspended in PBS and stored at − 20 °C for further experiments, or were resuspended in PB and stored at 4 °C for microscopic analyses.

For proteomic analyses, bacterial pellets were used to obtain the whole cell-extracts (fraction named BGAN8). For this, cell pellets were washed and concentrated 10-times in PBS, followed by cell-disruption in a French Press (Mini Cell Pressure Cell 3.7 for French Press G-M) at 2.5 kbar through three passages. After disruption, samples were centrifuged at 16,000*×g*, for 5 min at 4 °C to collect the supernatants which were kept at − 20 °C until their use. The protein content of both, isolated EVs and whole BGAN8 cell-extracts, was measured by Pierce BCA Protein Assay Kit (Thermo Fisher Scientific, Waltham, Massachusetts, USA) following the manufacturer’s instructions. These procedures were repeated three times (from three independent cultures of the BGAN8 strain).

### Microscopically analysis of *L. plantarum* BGAN8

Transmission electron microscopy (TEM) and scanning electron microscopy (SEM) of fresh cultures of *L. plantarum* BGAN8 were done at the “Scientific-technical services” of the University of Oviedo (Asturias), following standard procedures. Cryo-SEM was done at the “Electron Microscopy Service” of the Institute of Marine Sciences (ICM-CSIC, Barcelona). See supplementary material for detailed methodology.

### Microstructural analysis of EVs

Cryo-transmission electron microscopy (Cryo-TEM) analysis was performed with fresh (4 °C stored) EVs samples as described previously^38^. Briefly, one drop of the sample diluted 1/5 in milliQ water was applied on the carbon surface of a glow-discharged Lacey Carbon 300 mesh copper grid (Ted Pella, USA). The sample was allowed to adsorb for 1 min, at 100% humidity, inside the chamber Vitrobot Mark III (FEI Company, Eindhoven, Netherlands). The excess of liquid was automatically blotted with filter paper, followed by cryo-immobilization by plunge freezing in liquefied ethane. The vitrified sample was stored in liquid nitrogen until its observation in the cryo-TEM microscope. Plunge-frozen sample was transferred to a Tecnai F20 EM (FEI Companys) using a cryo-holder system (Gatan, Pleasanton, USA). The sample was examined at 200 kV, at a temperature ranging from − 179 to − 170 °C, using low-dose imaging conditions. Low-dose images were recorded with a 4096 × 4096-pixel CCD Eagle camera (FEI Company). This analysis was carried out at the Cryo-Electron Microscopy Unit of the Science and Technological Centres at the University of Barcelona (CCiT-UB).

### Protein fingerprinting

Protein profile of EVs and cell-extracts was analysed by 10% SDS-PAGE using 20 μg protein from each extract of the three replicates. Several bands were excised and identified by peptide fingerprint and fragmentation following comparison with the NCBIprot database (Inbiotec, Leon, Spain). In addition, PBS-extracts obtained in three independent biological replicates were mixed and analysed by liquid chromatography and mass spectrometry (Proteomic Unit, CAI Técnicas Biológicas, U.C.M. Madrid). In short, desalted protein digests were analysed by RP-LC–ESI–MS/MS in an EASY-nLC 1000 System coupled to the Q-Exactive HF mass spectrometer through the Nano-Easy spray source (all from Thermo Scientific, Mississagua, ON, Canada). Database search was performed against UniProt database (SwissProt and TrEMBL) with taxonomic restriction to *L. plantarum*. Detailed information of these procedures is described in the supplementary material.

### Internalization of EVs into HT29 cell line

The human colonic cell line HT29 (ATCC HTB-38) was obtained from the American Type Culture Collection and cultured in DMEM High Glucose (Dulbecco’s Modified Eagle Medium) supplemented with 10% (v/v) fetal bovine serum, 25 mM HEPES, 1% non-essential amino acids, penicillin (100 U/ml) and streptomycin (100 µg/ml). A total number of 1 × 10^5^ intestinal epithelial cells were seeded in 96-well plates suitable for fluorescence-based assays and let grown at 37 °C, 5% CO_2_, to 80% confluence.

EVs were fluorescently labeled with rhodamine isothiocyanate B-R18 (Molecular Probes, Thermo-Fisher Scientific) as previously described^46^ (see supplementary methods). Fluorescence of this probe is quenched in bilayer membranes at high concentration, but it is dequenched when probe is diluted after membrane fusion. Rhodamine-labeled EVs were suspended in PBS (0.2 M NaCl) containing a protease inhibitor cocktail and stored at 4ºC for up to 6 weeks.

To evaluate *L. plantarum* EVs uptake, HT29 cells were washed with PBS and incubated with rhodamine B-R18-labelled EVs (1 µg protein/well) suspended in DMEM without fetal bovine serum and phenol red. HT29 monolayers were incubated at 37ºC and fluorescence was measured over time, up to 5 h, in a Modulus-Microplate fluorescence reader (Ex 570 nm; Em 595 nm). Fluorescence intensity was normalized by fluorescence emitted by labeled-EVs in the absence of HT29 cells. To identify the endocytosis pathway, HT29 cells were pre-incubated for 1 h at 37 °C with the endocytosis inhibitors dynasore (80 μM), chlorpromazine (15 μg/ml,) filipin III (10 μg/ml) or nystatin (10 μg/ml), all reagents purchased from Sigma-Aldrich. Then, rhodamine B-R18-labeled EVs (1 μg protein/ well) were added to each well and cells were further incubated at 37 °C measuring the fluorescence over time. Control HT29 cells were not pre-treated with the inhibitors. Fluorometric uptake assays were repeated at least three independent times in triplicate.

Additionally, confocal fluorescence microscopy was used to confirm the entry pathway and the intracellular location of labeled-EVs^46^. HT29 cells, grown in 8-well chamber slide (Ibidi GmbH, Martinsried, Germany) to 80% confluence, were incubated with rhodamine B-R18-labeled EVs (2 μg/ well) at 37 °C. After 1 h and 4 h, cells were washed with PBS and the nuclei were labeled with DAPI whereas membranes were labeled with Alexa Fluor-488 wheat germ agglutinin (WGA, Molecular Probes) (1 μg/ml, 4 °C), after incubation for 25 min, followed by fixation for 30 min with 3% paraformaldehyde. Parallel experiments were performed with HT29 cells pre-incubated with dynasore (80 μM) or filipin III (10 μg/ml). Fluorescence microscopy was carried out with a Leica TCS SP5 laser scanning confocal spectral microscope, using the 63 × oil immersion objective lens. Images were captured with a Nikon color camera (16 bit). Fluorescence was recorded at 405 nm (blue, DAPI), 488 nm (green, WGA) and 546 nm (red; rhodamine B-R18). Z-stack images were taken at 0.5 to 1.0-μm. The Fiji image processing package was used for fluorescence imaging analysis.

### Monitoring the effect of EVs on HT29 cell line

The behaviour of HT29 cells in the presence of EVs or UV-inactivated *L. plantarum* BGAN8 bacteria was monitored using the real time cell analyser (RTCA-DP) xCelligence (ACEA Bioscience Inc., San Diego, CA, USA) as previously described^49^. In short, 16-well E-plates (ACEA Bioscience) were seeded (2 × 10^5^ cells in 100 μl culture medium) and placed into the monitoring device, which records the impedance signal, to reach a stable value indicating the formation of a monolayer. At this point (about 22 ± 1 h of incubation), supernatants were removed and 200 μl culture medium containing different concentrations of EVs (from 0.63 μg/ml to 40 μg/ml protein content) or UV-treated bacteria (ratio 10:1, 50:1 and 100:1, bacteria: HT29 cells) was added. The incubation was monitored for additional 38 h and the impedance data were normalized by the control sample (HT29 incubated in the culture medium alone) and by the time at which the EVs or inactivated bacteria were added.

### Statistical analyses

Statistical analysis was performed using SPSS (version 20.0, Chicago, IL, USA) software package. Quantitative data are presented as mean ± standard error. Differences among treatment groups were assessed using one-way ANOVA followed by Tukey’s test (*p* value < 0.05).

## Supplementary Information

Supplementary Dataset.

Supplementary Information.

## Data Availability

Data generated or analysed during this study, as well as detailed protocols and methods, have been made available in the supplementary section of this article. If additional data presented here is required, it will be made available upon request to the corresponding author.
