# Supplementary material for: Proteomic profile of extracellular vesicles released by Lactiplantibacillus plantarum BGAN8 and their internalization by non-polarized HT29 cell line
Source: Sci Rep. 2020 Dec 11;10:21829. doi: 10.1038/s41598-020-78920-z (PMC7732981; doi:10.1038/s41598-020-78920-z)

Supplementary information

Proteomic profile of extracellular vesicles released by *Lactiplantibacillus plantarum* BGAN8 and their internalization by non-polarized HT29 cell line

Author list

Svetlana Sokovic-Bajic[1,3], Maria-Alexandra Cañas[4,5‡] , Maja Tolinacki[4], Josefa Badia[4,5], Borja Sánchez[1,2], Natasa Golic[3], Abelardo Margolles[1,2], Laura Baldomá[4,5] and Patricia Ruas-Madiedo[1,2*]

Affiliations

[1,] Department of Microbiology and Biochemistry of Dairy Products, Instituto de Productos Lácteos de Asturias – Consejo Superior de Investigaciones Científicas (IPLA-CSIC), Villaviciosa, Asturias, Spain

[2,] Group Funcionality and Ecology of Beneficial Microbes, Instituto de Investigación Sanitaria del Principado de Asturias, (ISPA), Oviedo, Asturias, Spain

[3,] Laboratory for molecular microbiology, Institute of Molecular Genetics and Genetic Engineering (LMM-IMGGE), University of Belgrade, Belgrade, Serbia

[4,] Secció de Bioquímica i Biología Molecular, Departament de Bioquímica i Fisiologia, Facultat de Farmàcia i Ciències de l'Alimentació, Universitat de Barcelona, Barcelona, Spain.

[5,] Institut de Biomedicina de la Universitat de Barcelona (IBUB), Institut de Recerca Sant Joan De Déu (IR-SJD), Barcelona, Spain.

‡ Present address: Laboratorio de Endocarditis Experimental, Hospital Clinic – Institut d'Investigacions Biomèdiques August Pi i Sunyer (IDIBAPS)

*** corresponding author: P. Ruas-Madiedo ruas-madiedo@ipla.csic.es**

Methods

1. Electron microscopy techniques

For transmission electron microscopy (TEM) analysis, cultures of *L. plantarum* BGAN8 (grown in MRS for 20 h) were washed twice with PBS (at 10,000*xg* for 10 min) and resuspended in PB. The bacterial suspension was fixed in PB containing 4% glutaraldehyde and 2% paraformaldehyde for 3 h, and sent to the "Servicios Científico Técnicos" (SCT, scientific-technical services) of the University of Oviedo (Oviedo, Asturias, Spain) for visualization on the JEOL-2000 EX-II TEM (Jeol Ltd., Tokyo, Japan), after sample preparation following standard procedures. For scanning electron microscopy (SEM) 10 µl of this bacterial suspension (concentrated 10-times and fixed) were mounted in Formavar-carbon Cu 300 mesh girds, before gold coating. The analysis was done with the JEOL-6610LV SEM (Jeol Ltd.) microscope at the same SCT of the Oviedo University.

The cryo-SEM analysis was performed as previously reported [1]. In short, one drop of the bacterial suspension, obtained from a 20-h culture washed and concentrated 10-times in Ringer (Merck, Darmstadt, Germany), were placed on a special cryo stub. They were then immediately plunged into liquid nitrogen slush in the cryogen unit. Once samples were frozen, they were transferred under vacuum to the cryo preparation chamber Quorum PP3000T (Quorum Technologies, www.quorumtech.com), attached at the SEM column. The frozen samples were fractured with a cooled adjustable knife. After this, were sublimed at −90ºC for 5 min, sputter coated with Pt for 30 s and transferred to a cold stage inside the chamber of the Hitachi S-3500N SEM (Hitachi High-Technologies Corporation, Tokyo, Japan, www.hitachi-hightech.com). The samples were maintained at −135ºC during the observation that was made at an acceleration voltage of 4 kV. The cryo-SEM was performed at the Electron Microscopy Service of the Institute of Marine

Sciences (ICM-CSIC, Barcelona, Spain). We acknowledge the excellent technical assistance of Jose Manuel Fortuño Alós (ICM-CSIC).

2. Protein fingerprinting

2.1. Peptide mass fingerprinting (MALDI TOF/TOF)

The protein profiles of EVs and BGAN8 cell-free extracts were analysed by 10% SDS-PAGE using 20 µg protein from each extract. Differential bands were excised and sent to the "Instituto de Biotecnología de León" (Inbiotec, León, Spain) for protein identification through a standard peptide mass fingerprinting protocol. Bands were resuspended in 50 µL of 50 mM NH_4HCO_3, and reduced in the presence of DTT 5.8 mM (GE-Healthcare, Chicago, IL) during 5 minutes at 95ºC. Samples were further alkylated using iodoacetamide 11.7 mM (GE-Healthcare) during 15 min at RT and in the dark. Trypsin (Sequencing Grade Modified Trypsin, Promega, Madison, WI) was added at a final concentration of 10 ng/mg sample, and the mixture was kept overnight at 37ºC. Finally, peptides were centrifuged at 14,000*xg* to remove polyacrylamide debris during 10 minutes and supernatants were dried in a vacuum device. Peptides were directly dissolved in 5 µl of a matrix solution, composed of a saturated mixture of alpha-cyano-4-hydroxy cinnamic acid (Sigma-Aldrich), in 40 % (v/v) acetonitrile and 0.1 % (v/v) trifluoroacetic acid. The samples (1 µl) were subjected to mass analyses on a 4800 MALDI TOF/TOF mass spectrometer (Applied Biosystems, Foster City, CA). Proteins were identified by comparison against the NCBIprot non-redundant database.

2.2. Liquid chromatography and mass spectrometry analysis (LC/MS)

This analysis was carried out in the external service of the Proteomics Unit from the Complutense University of Madrid (UCM, Madrid, Spain), a member of National

Institute of proteomic (proteored-ISCIII, Madrid) supported by “grant PT17/0019” of the PE I+D+i 2013-2016, and the “grant PRB3 (IPT17/0019)” of the ISCIII-SGEFI / ERDF. Enrique Pérez and Felipe Clemente, from the Proteomics unit, are acknowledged for the technical support.

For sample preparation, lyophilised EVs or whole BGAN8 cell-extracts were resuspended in water and the three triplicates of each, obtained from three independent cultures of *L. platarum* BGAN8, were mixed. Protein content of the two samples was quantified by Bradford method and 50 μg were loaded in a 4% staking-10% running SDS-PAGE gel. Electrophoresis was stopped 3 mm above the running gel, just before starting the separation. The stacking gel was stained with colloidal Coomassie and the protein band was cut to carry out an in-gel trypsin digestion. In short, proteins in-gel were reduced with DTT, alkylated with iodacetamide and digested with a 1/25 (w/w) ratio of recombinant trypsin (trypsin sequencing grade; Roche, Basilea, Switzerland) overnight at 37ºC. After digestion, peptides were desalted and concentrated with a reverse phase C18 chromatographic column (OMIX C18, Agilent Technologies, Santa Clara, CA) using 80% acetonitrile / 0.1% trifluoroacetic acid as mobile phase. Concentrated samples were freeze-dried in Speed-vac, resuspended in 2% acetonitrile, 0.1% formic acid and stored at -20ºC before analysis.

The peptide fingerprint analysis was carried out as previously described by Rico-San Román et al. (2020) [2] using an EASY-nLC 1000 System coupled to the Q-Exactive HF mass spectrometer through the Nano-Easy spray source (all from Thermo Scientific, Mississagua, ON, Canada). Samples were loaded onto an Acclaim PepMap 100 Trapping column (Thermo Scientific, 20mm x 75 μm ID, 3 μm C18 resin with 100 Å pore size) using buffer A (mobile phase A: 2% acetonitrile, 0.1% formic acid); then, they were separated and eluted on a C18 resin analytical column NTCC (Nikkyo Technos Co., Ltd.,

Tokyo, Japan) of 150 mm x 75 μm ID, 3 μm C18 resin with 100 Å pore size, with an integrated spray tip. A 120-minute gradient of 5% to 35% buffer B (100% acetonitrile, 0.1% formic acid) in buffer A, at a constant flow rate of 250 nl/min, was used. Data acquisition was performed with a Q-Exactive HF, using an ionspray voltage 1.8 Kv and ion transfer temperature of 250ºC, by means of the Xcalibur 4.0 software (Thermo Scientific) with data-dependent acquisition (DDA) and in positive mode. For MS2 scan, the top 15 most abundant precursors with charges of 2 to 4+ in MS1 scans, for higher energy collisional dissociation (HCD) fragmentation with a dynamic exclusion of 20 s, were selected. The MS1 scans were acquired at m/z range of 350–2000 Da with mass resolution of 60,000 and automatic gain control (AGC) target of 3E6, at a maximum Ion Time (ITmax) of 60 ms. The threshold to trigger MS2 scans was 2E3; the normalized collision energy (NCE) was 27%; the resolved fragments were scanned at mass resolution of 30,000 and AGC target value of 1E5 in a ITmax of 100ms.

Peptide identification from raw data was carried out according to Rico-San Román et al. (2020), using Mascot v. 2.6.1 search engine, the Protein Discoverer 2.2 Software (Thermo Scientific), and a database search against UniProt database (SwissProt and TrEMBL) with taxonomic restriction to *L. plantarum* (52,531 sequences and a contaminant database of 247 sequences). The following parameters were used for the searches: tryptic cleavage after Arg and Lys, up to two missed cleavage sites allowed, and tolerances of 10 ppm for precursor ions and 0.1 Da for MS/MS fragment ions; the searches were performed allowing optional methionine oxidation and methionine loss plus acetyl protein N-terminal and fixed carbamidomethylation of cysteine. Search against decoy database (integrated decoy approach) was used to FDR calculate. The Mascot Scores were adjusted by a percolator algorithm. The acceptances criteria for

proteins identification were a FDR < 1% and at least one peptides identified with high confidence (CI>95%).

3. EVs labelling protocol

The EVs labelling was performed as previously reported by Cañas et al. (2016) [3]; in short, isolated EVs were washed with PBS, resuspended in labelling buffer (50 mM Na_2CO_3, 100 mM NaCl, pH 9.2) in the presence of 1 mg/ml octadecyl rhodamine B-R18 and incubated for 1 h at 25°C. Labelled EVs were pelleted by centrifugation at 150,000*xg* for 1 h at 4ºC, resuspended in PBS (0.2 M NaCl) and washed twice to fully remove the unbound dye. After a final centrifugation step, the rhodamine-labelled EVs were resuspended in PBS (0.2 M NaCl) containing a protease inhibitor cocktail (Complete Protease Inhibitor Tablet, Roche) and stored at 4ºC for up to 6 weeks.

References

[1]. Bachtarzi, H., Speciale, I., Kharroub, K., De Castro, C., Ruiz, L. & Ruas-Madiedo, M. Selection of exopolysaccharide-producing *Lactobacillus plantarum* (*Lactiplantibacillus plantarum*) isolated from Algerian fermented foods for the manufacture of skim-milk fermented products. *Microorganisms* **8**, 1101 (2020).

[2]. Rico-San Román, L. et al. Comparative tachyzoite proteome analyses among six *Neospora caninum* isolates with different virulence. *International Journal for Parasitology* **50**, 377–388 (2020).

[3]. Cañas, M. A. et al. Outer membrane vesicles from the probiotic Escherichia coli Nissle 1917 and the commensal ECOR12 enter intestinal epithelial cells via clathrin-dependent endocytosis and elicit differential effects on DNA damage. PLoS ONE **11**, e0160374 (2016).

Supplementary figures

Fig. S1. *L. plantarum* BGAN8 visualized under transmission electron microscope JEOL-2000 EX-II (Jeol Ltd., Tokyo, Japan). Visualization performed at the service of "Servicios Científico Ténicos", Universidad de Oviedo (Asturias, Spain).

Fig. S2. *L. plantarum* BGAN8 visualized under scanning electron microscope JEOL-6610LV (Jeol Ltd., Tokyo, Japan). Visualization performed at the service of "Servicios Científico Ténicos", Universidad de Oviedo (Asturias, Spain).

Fig. S3. *L. plantarum* BGAN8 visualized under cryo-scanning electron microscope Hitachi S-3500N (Hitachi High-Technologies Corporation, Tokyo, Japan). Visualization performed at the Electron Microscopy Service, Institute of Marine Sciences (ICM-CSIC, Barcelona, Spain).

Fig.S4. Sediment of extracellular vesicles in the bottom of an ultra-centrifuge tube obtained after purification from the cell-free supernatant of *L. plantarum* BGAN8 cultured in MRS medium.

Fig. S5. Visualization of EVs from *L. plantarum* BGAN8 under cryo-scanning electron microscope Hitachi S-3500N (Hitachi High-Technologies Corporation, Tokyo, Japan). Visualization performed at the Electron Microscopy Service, Institute of Marine Sciences (ICM-SCIS, Barcelona, Spain).

Fig. S6. Extracellular vesicles from *L. plantarum* BGAN8 visualized under cryo-transmission electron microscope Tecnai F20 (FEI Company, Eindhoven, Netherlands). Visualization performed at the service of Crio-Microscòpia Electrònica, Centres Científics i Tecnològics, Universitat de Barcelona (Barcelona, Spain).

Fig. S7. Monitoring the behaviour of HT29 monolayers by measuring the impedance signal (Cell Index) in the RTCA-DP xCelligence (ACEA Bioscience Inc., San Diego, CA). Monolayer formation was monitored for 22 h before the addition of the EVs or the

whole cell-extracts from *L. plantarum* BGAN8; afterwards the HT29 behaviour was monitored for additional 38 h.

Fig. S8. Monitoring in real time the acidification curve of *L. plantarum* BGAN8 using the ORION Versa Star (Thermo Scientific Inc. USA). At defined sampling points the OD_{600nm} was determined in the Bio Photometer Plus (Eppendorf, Hamburg, Germany) and counts obtained after plating serial dilutions (in Ringer, Merck) of the samples on agar-MRS (Biokar Diagnostics, Beauvais, France) followed by incubation at 30ºC for 48 hours.

Supplementary datasheet

"Sokovic-Bajic et al.-Database of protein fingerprint.xlsx": database of protein fingerprint.xlsx: LC/MS protein datasheets. Analysis carried out in the external service of the Proteomics Unit from the Complutense University of Madrid, (UCM, Madrid, Spain).

Fig. S1

TEM (Transmission Electron Microscopy)

***Lactiplantibacillus plantarum* BGAN8**

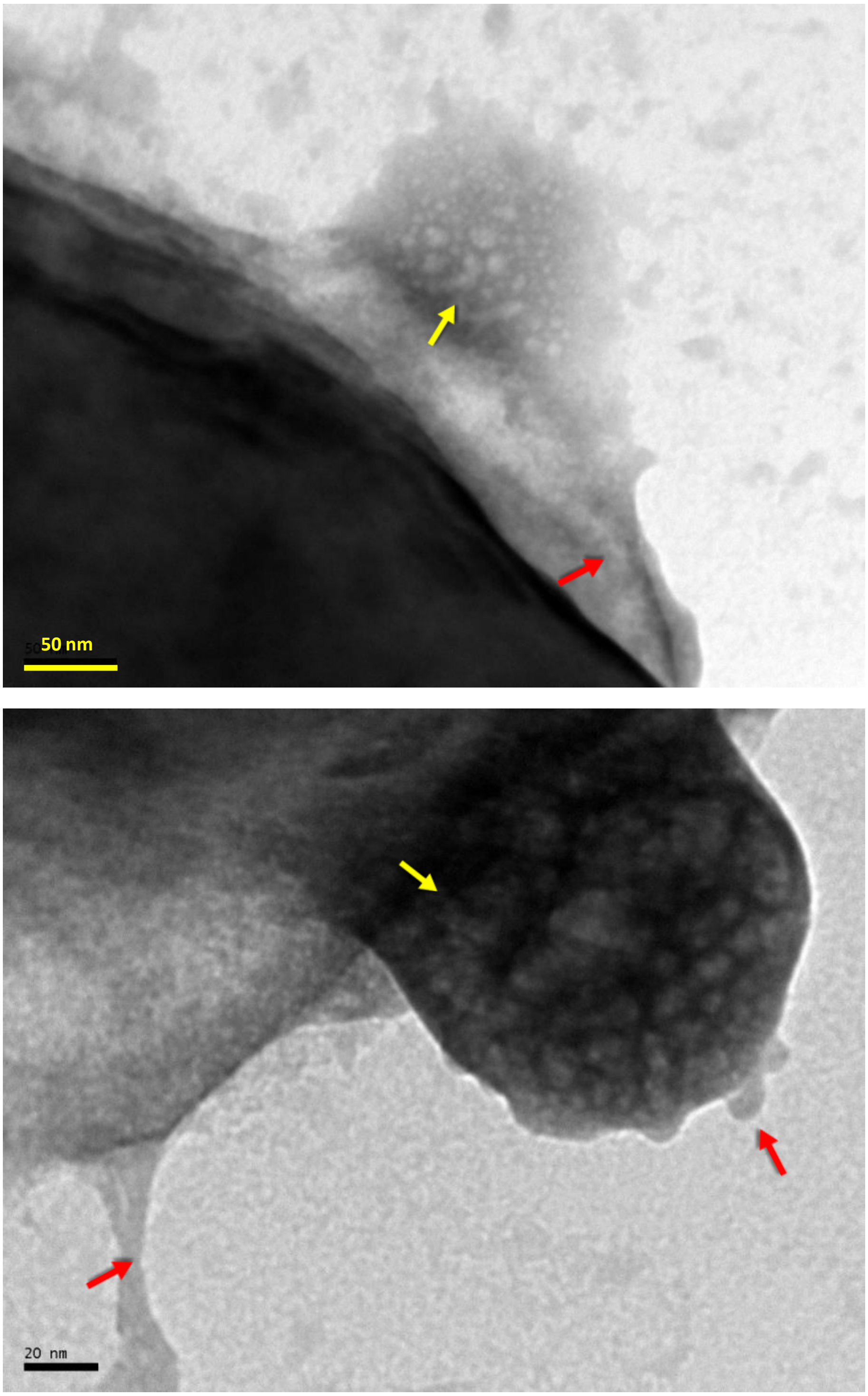

Red arrow: EPS covering the *L. plantarum* BGAN8; Yellow arrow: EVs

Fig. S2

SEM (Scanning Electron Microscopy)

Lactiplantibacillus plantarum **BGAN8**

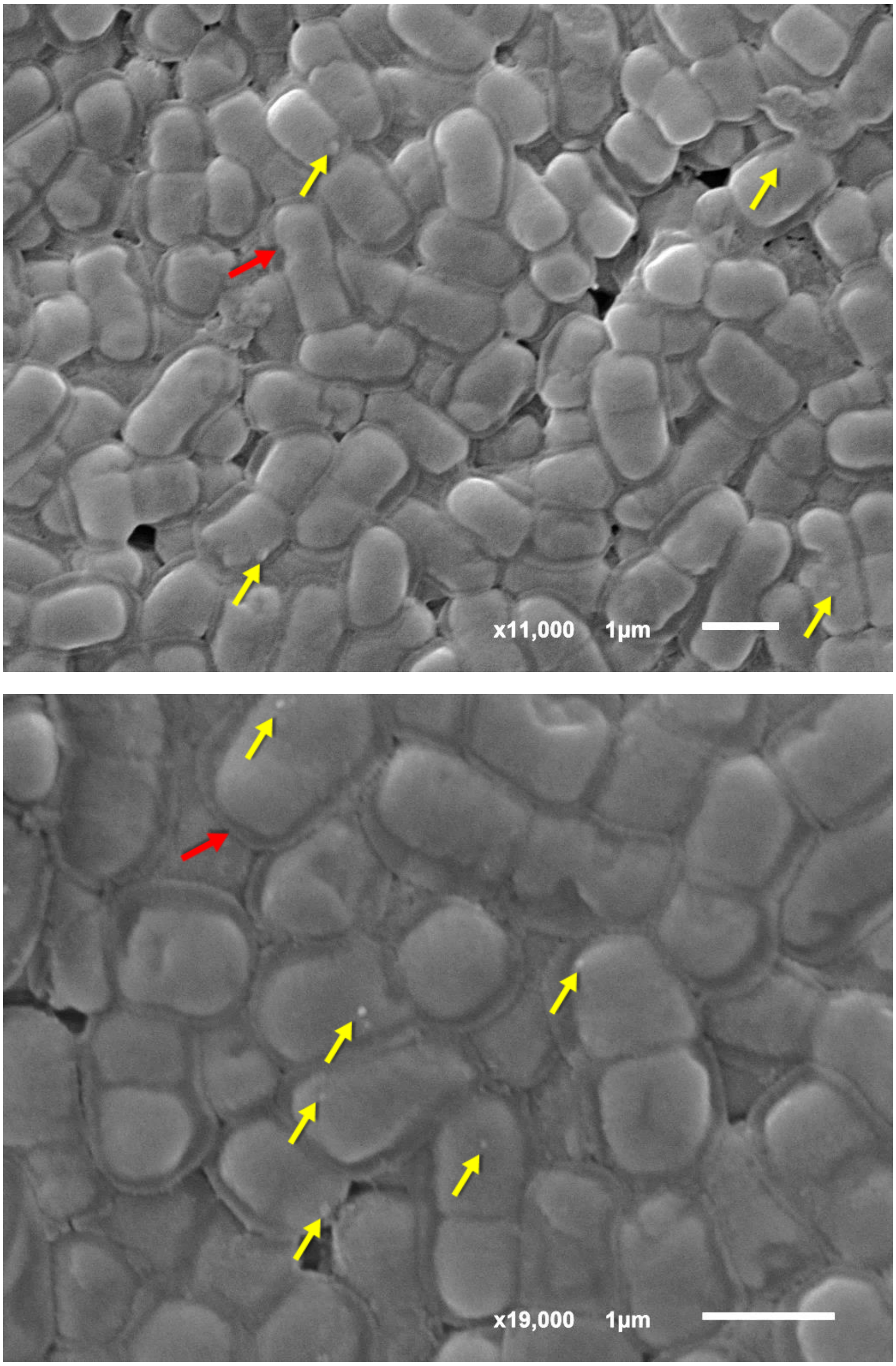

Red arrow: EPS covering the L. plantarum BGAN8; Yellow arrow: EVs

Fig. S3

Cryo-SEM (cryo-Scanning Electron Microscopy)

Lactiplantibacillus plantarum **BGAN8**

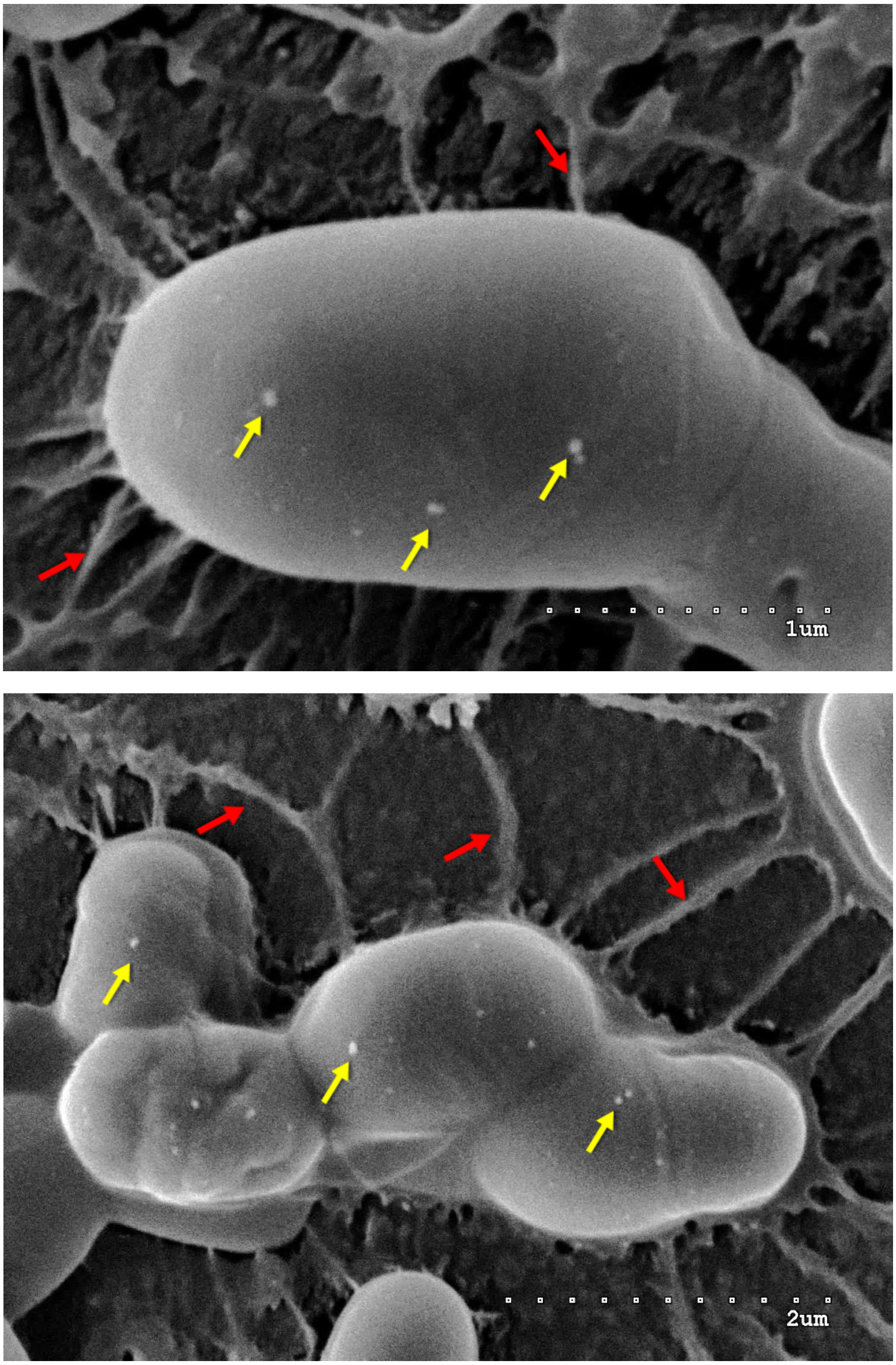

Red arrow: EPS covering the L. plantarum BGAN8; Yellow arrow: EVs

Fig. S4

Sediment of EVs purified from
***Lactiplantibacillus plantarum* BGAN8**

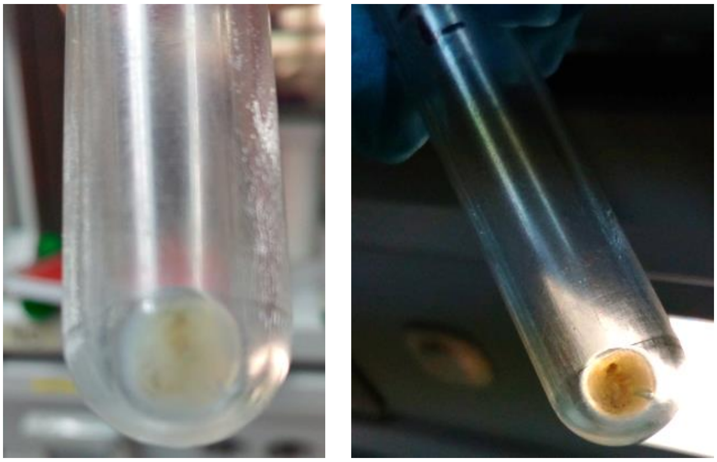

Fig. S5

Cryo-SEM (cryo-Scanning Electron Microscopy)

EVs from *Lactiplantibacillus plantarum* BGAN8

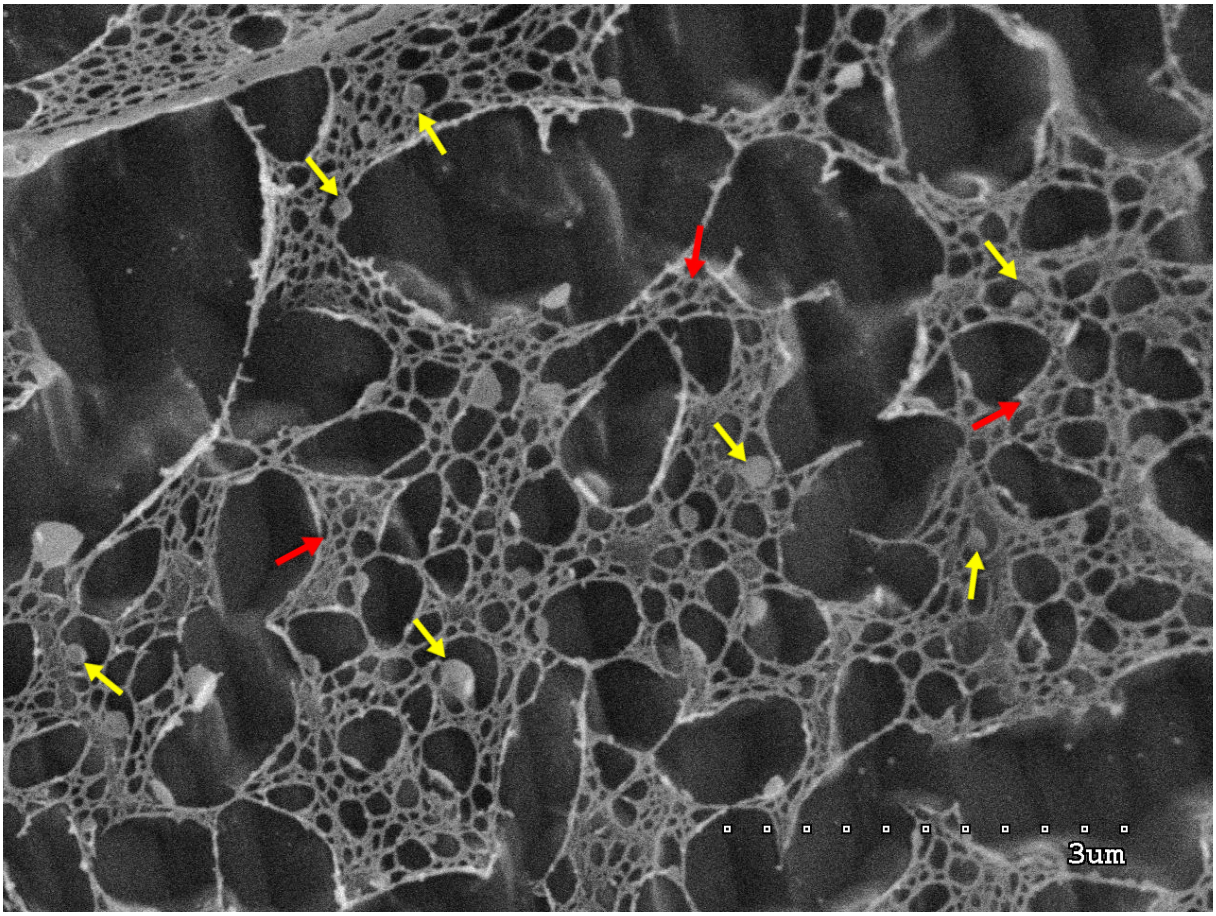

Red arrow: EPS; Yellow arrow: EVs

Fig. S6

Cryo-TEM (cryo-Transmission Electron Microscopy)

EVs from *Lactiplantibacillus plantarum* BGAN8

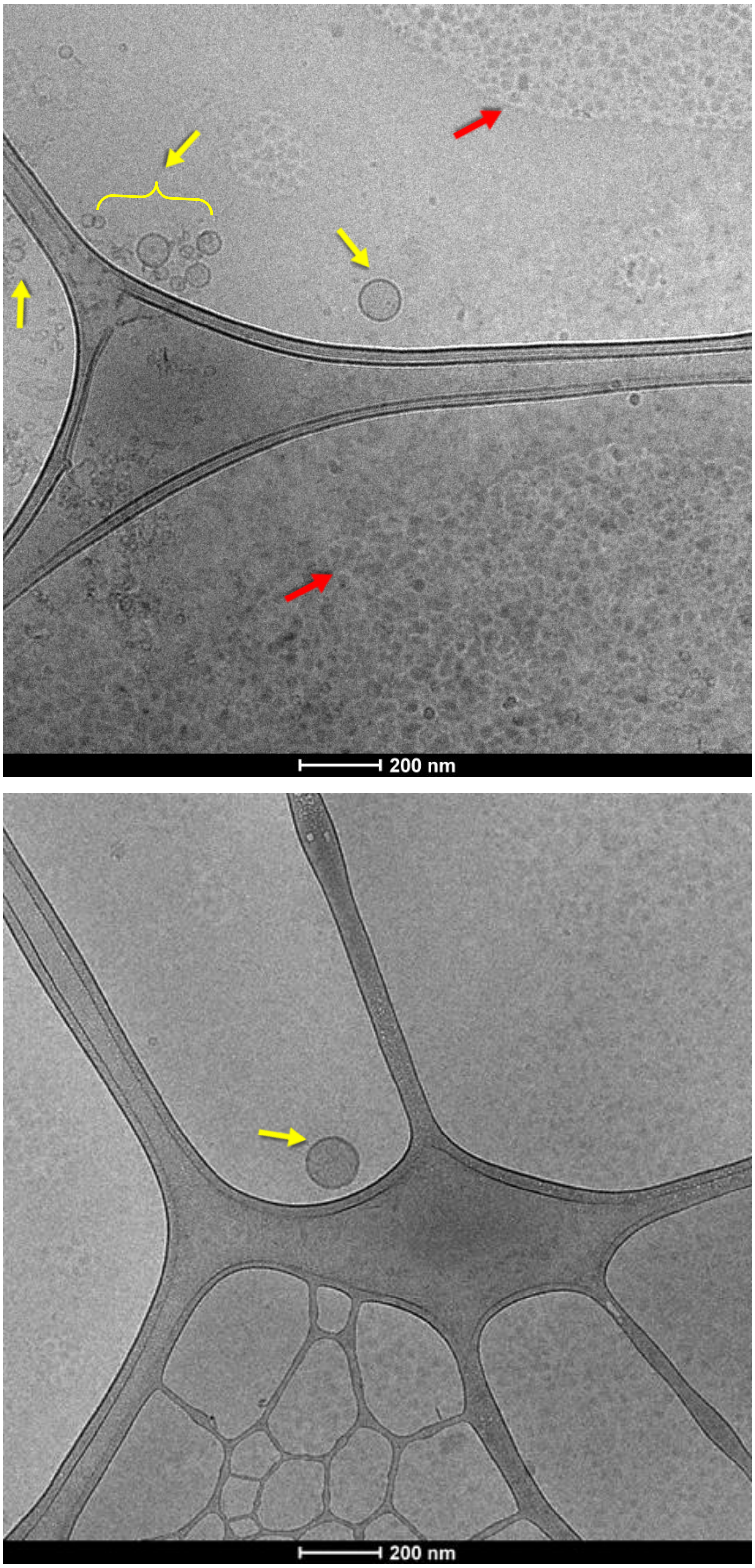

Red arrow: EPS; Yellow arrow: EVs

Fig. S7

RTCA (Real Time Cell Analyzer)

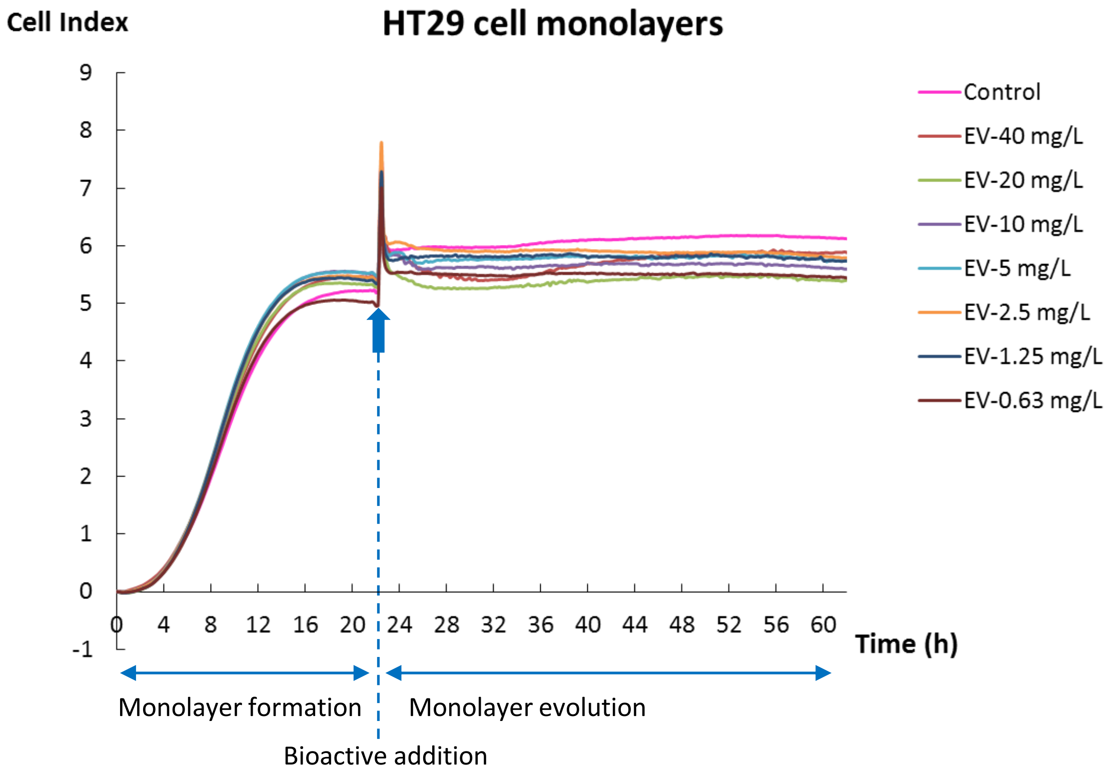

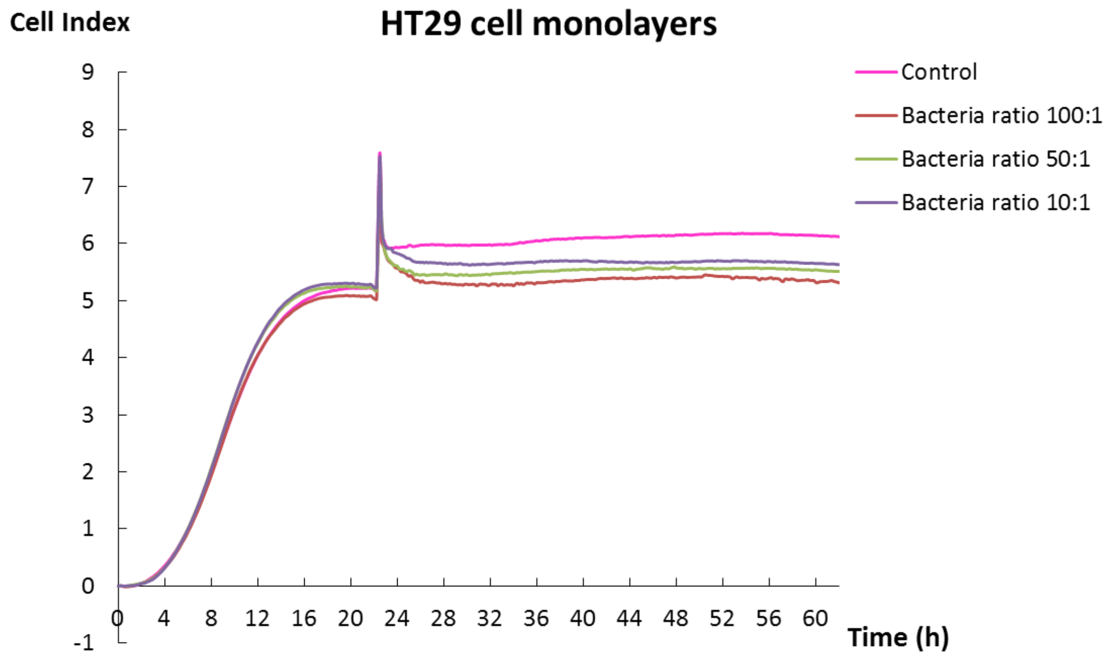

Fig. S8

Growth of *Lactiplantibacillus plantarum* BGAN8 in MRS

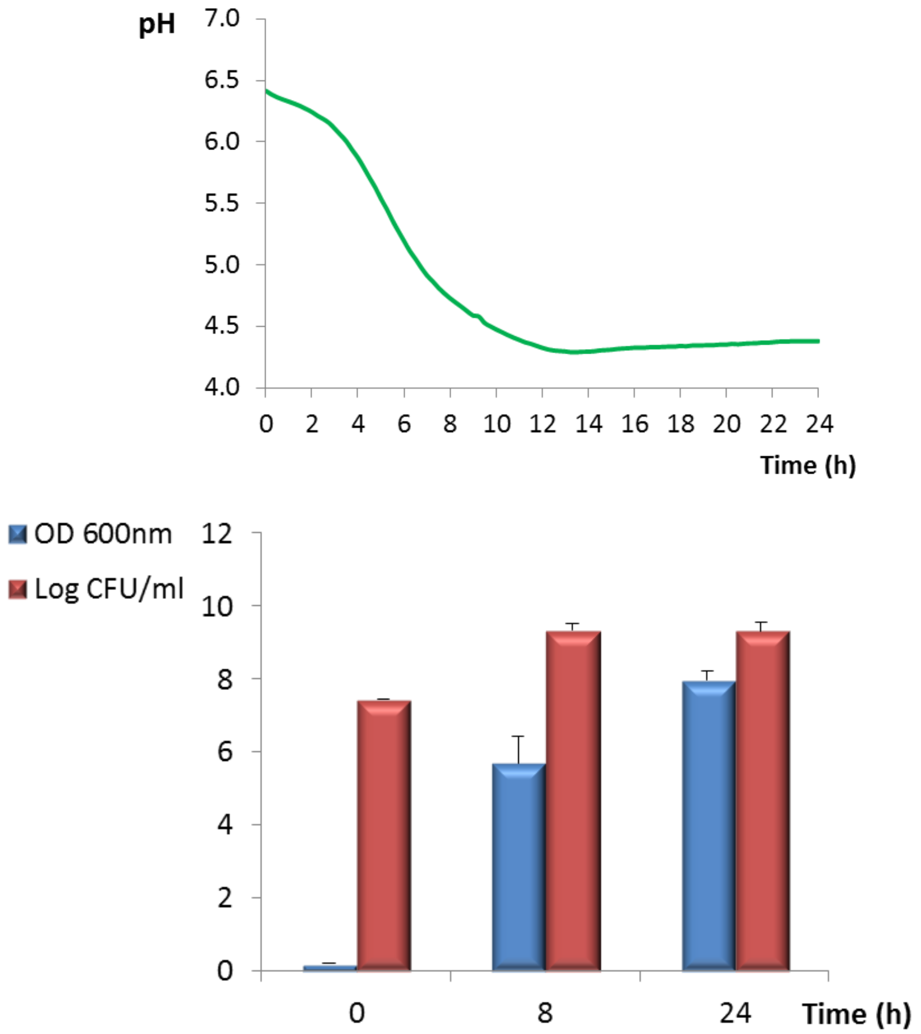

Original gel corresponding to Figure 3. Proteomic fingerprint of the whole cell-extract and the EVs from *Lactiplantibacillus plantarum* BGAN8. (A) SDS-PAGE profile of three culture replicates (A, B, and C) of EVs and BGAN8 whole-extract.

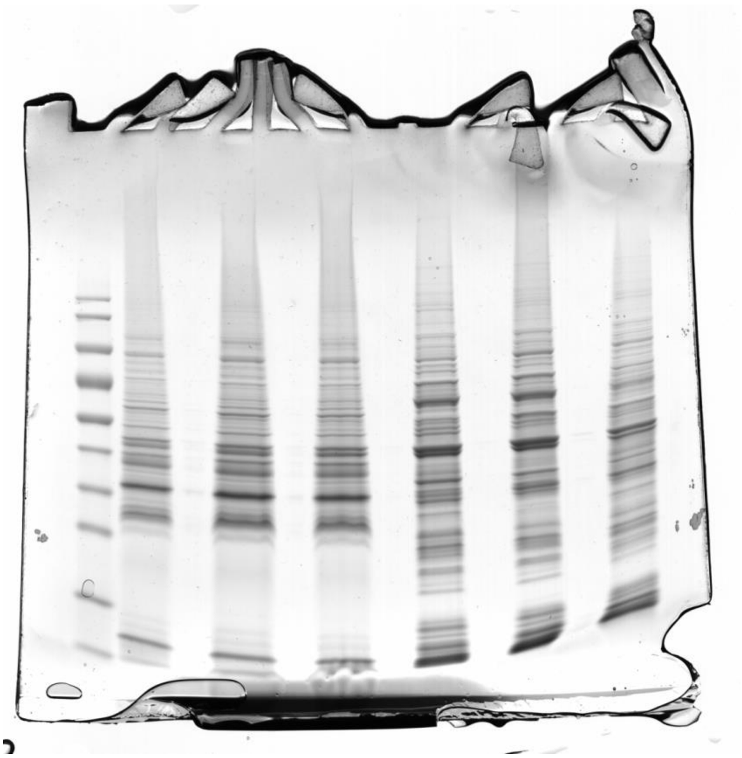

Supplement: Supplementary file 2 — Supplementary Information. [file 41598_2020_78920_MOESM2_ESM.pdf]
